# PIK3CA mutation in endometriotic epithelial cells promotes viperin-dependent inflammatory response to insulin

**DOI:** 10.1186/s12958-023-01094-6

**Published:** 2023-05-11

**Authors:** Mike R. Wilson, Shannon Harkins, Jake J. Reske, Rebecca A. Siwicki, Marie Adams, Victoria L. Bae-Jump, Jose M. Teixeira, Ronald L. Chandler

**Affiliations:** 1 Department of Obstetrics, Gynecology and Reproductive Biology, College of Human Medicine, Michigan State University, Grand Rapids, MI 49503 USA; 2 Genomics Core Facility, Van Andel Research Institute, Grand Rapids, MI 49503 USA; 3 Lineberger Comprehensive Cancer Center, University of North Carolina at Chapel Hill, Chapel Hill, NC 27599 USA; 4 Division of Gynecologic Oncology, University of North Carolina at Chapel Hill, Chapel Hill, NC 27599 USA; 5 Reproductive and Developmental Sciences Program, Michigan State University, East Lansing, MI 48824 USA; 6 Department for Epigenetics, Van Andel Research Institute, Grand Rapids, MI 49503 USA

**Keywords:** Insulin, Phosphatidylinositide 3-kinase, Endometrium, Endoplasmic reticulum stress, Interferon

## Abstract

Endometrial epithelia are known to harbor cancer driver mutations in the absence of any pathologies, including mutations in PIK3CA. Insulin plays an important role in regulating uterine metabolism during pregnancy, and hyperinsulinemia is associated with conditions impacting fertility. Hyperinsulinemia also promotes cancer, but the direct action of insulin on mutated endometrial epithelial cells is unknown. Here, we treated 12Z endometriotic epithelial cells carrying the PIK3CA^H1047R^ oncogene with insulin and examined transcriptomes by RNA-seq. While cells naively responded to insulin, the magnitude of differential gene expression (DGE) was nine times greater in PIK3CA^H1047R^ cells, representing a synergistic effect between insulin signaling and PIK3CA^H1047R^ expression. Interferon signaling and the unfolded protein response (UPR) were enriched pathways among affected genes. Insulin treatment in wild-type cells activated normal endoplasmic reticulum stress (ERS) response programs, while PIK3CA^H1047R^ cells activated programs necessary to avoid ERS-induced apoptosis. PIK3CA^H1047R^ expression alone resulted in overexpression (OE) of Viperin (RSAD2), which is involved in viral response and upregulated in the endometrium during early pregnancy. The transcriptional changes induced by insulin in PIK3CA^H1047R^ cells were rescued by knockdown of Viperin, while Viperin OE alone was insufficient to induce a DGE response to insulin, suggesting that Viperin is necessary but not sufficient for the synergistic effect of PIK3CA^H1047R^ and insulin treatment. We identified interferon signaling, viral response, and protein targeting pathways that are induced by insulin but dependent on Viperin in PIK3CA^H1047R^ mutant cells. These results suggest that response to insulin signaling is altered in mutated endometriotic epithelial cells.

**Supplementary Information:**

The online version contains supplementary material available at 10.1186/s12958-023-01094-6.

## Introduction

PIK3CA is a component of the Phosphoinositide 3-kinase (PI3K), an enzyme that regulates cellular growth and proliferation. PIK3CA (also called p110α) is the catalytic subunit of PI3Kα. Components of the PI3K-AKT-mTOR pathway are regularly mutated across cancer, with PIK3CA among the most frequently mutated genes [1]. PIK3CA mutations occur most commonly in endometrial cancer (EC) relative to other cancers [1], and most ECs have a mutation in the PI3K pathway [2, 3]. PIK3CA mutations are also observed in both endometriosis and endometriosis-associated ovarian cancer, such as ovarian clear-cell carcinoma, conditions thought to be derived from abnormal endometrial epithelium [4–9]. Furthermore, PIK3CA mutations have been observed at high frequency in normal endometrial epithelial cells without cancer or endometriosis [4, 10, 11]. PIK3CA mutant endometrial epithelial cells can expand clonally within an epithelial gland, while unique PIK3CA mutations are observed among distinct glands in the same uterus [4, 10, 11].

Insulin is a peptide hormone secreted by the pancreas that regulates glucose homeostasis, and high levels of circulating insulin can activate the PI3K-AKT-mTOR pathway which promotes growth in cancer cells [12]. Insulin secretion increases during early pregnancy [13], during which time it directly activates glycogen synthesis in endometrial glands [14]. Hyperinsulinemia is a common feature among conditions that impact fertility, including obesity [15], type 2 diabetes mellitus [16] and polycystic ovarian syndrome (PCOS) [17]. Among women with PCOS, those with pregnancy complications had elevated serum insulin levels compared to those without pregnancy complications [18]. Endometria in women with PCOS have impaired glucose transport and utilization, chronic low-grade inflammation, and immune dysfunction [17]. PCOS and endometriosis can co-occur in women, and PCOS increases endometrial cancer risk [19–21]. Hyperinsulinemia is also an independent risk factor for EC [12] and ovarian cancer [22], although associations between different types of ovarian cancer and hyperinsulinemia are less understood.

Give the links between abnormal endometrium and conditions associated with hyperinsulinemia, we hypothesized that insulin may have a direct impact on endometrial epithelia, and that the response to insulin would be altered in cells that have acquired a PIK3CA mutation. We identified a unique response to insulin dependent on PIK3CA mutation, leading to changes in ERS and interferon response. These gene expression alterations were driven by aberrant expression of Viperin [23], an interferon-inducible protein upregulated in the endometrium during early pregnancy [24], in PIK3CA-mutant cells. Knockdown of Viperin rescued this effect, while Viperin OE was insufficient, implicating a unique role for Viperin in the context of PIK3CA-mutant, insulin responsive cells. These results suggest that the effects of insulin are augmented in the presence of PIK3CA mutation as a result of Viperin overexpression.

## Methods

### Cell lines and treatments

12Z immortalized human endometriotic epithelial cells [25] were maintained in DMEM/F12 media (BioWest, cat# L0091) supplemented with 10% fetal bovine serum (FBS), 1% L-glutamine, 1% penicillin/streptomycin (P/S), 15 mM HEPES and 3.151 g/L D-glucose. For transfections, 12Z cells were seeded at 40,000 cells/mL in media without P/S. After 24 h (hrs), cells were transfected with 50 pmol/mL of siRNA (Dharmacon, ON-TARGETplus Non-targeting Pool, human RSAD2 #91,543 SMARTpool) using RNAiMax (ThermoFisher) at a volumetric ratio of 1:1 vol:vol in OptiMEM (Gibco). After 24 h, cells were transfected with 1,000 ng/mL with pBabe PIK3CA^H1047R^, pBabe empty vector, pCAG Viperin or pCAG empty vector using FuGene HD transfection reagent (Promega) according to the manufacturers’ instructions at 2:1 volume:mass. Media was replaced after 4 h. The pBabe PIK3CA^H1047R^ plasmid was a gift from Jean Zhao (Addgene plasmid 12,524) [26]. The pCAG Viperin plasmid was a gift from Ella Sklan [27]. The following day, serum was removed from the media, and after 4 h 100 nM insulin (Sigma-Aldrich, cat# 19,278) or vehicle (1:1000 dilution of 0.9% saline, 0.02% BSA) was added. Cells were collected 24 h post-treatment for downstream analysis. For cell growth measurements, cells were treated with tunicamycin concentrations ranging from 1 nM to 100 nM for 48 h, and cell density was measured by incubation with 2 µg/mL calcein-AM for 1 h and fluorescence measurement using a SpectraMax i3x (Molecular Devices).

### Western blotting

Whole cell lysates were collected in RIPA (Cell Signaling). Protein was quantified using Micro BCA Protein Assay Kit (ThermoFisher) and FlexSystem3 plate reader. Samples were run on a 4–15% gradient SDS-PAGE gel (BioRad) and transferred to PVDF membrane using TransBlot Turbo (BioRad). Antibodies were used at these dilutions: 1:1000 AKT (4691, Cell Signaling); 1:1000 β-Actin (8457, Cell Signaling); 1:2000 Phospho-AKT (Ser473) (4060, Cell Signaling); 1:1000 Viperin (MABF106, Millipore); 1:2000 Anti-rabbit IgG, HRP-linked Antibody (7074, Cell Signaling) and 1:2000 Anti-mouse IgG, HRP-linked Antibody (7076, Cell Signaling). Exposures were obtained using Clarity Western ECL Substrate (BioRad) and ChemiDoc XRS + imaging system (BioRad). Uncropped images are available in Supplemental Fig. 2.

### mRNA-seq library construction, sequencing and analysis

RNA-seq was performed on n = 3 independent experiments for each condition. Libraries were prepared by the Van Andel Genomics Core from 500 ng of total RNA using KAPA mRNA HyperPrep kit (v4.17) (Kapa Biosystems). RNA was sheared to 300–400 bp. Prior to PCR amplification, cDNA fragments were ligated to IDT for Illumina unique dual adapters (IDT DNA Inc). Quality and quantity of libraries were assessed using Agilent DNA High Sensitivity chip (Agilent) and QuantiFluor® dsDNA System (Promega). Individually indexed libraries were pooled and 50 bp, paired end sequencing was performed using a Illumina NovaSeq6000 sequencer and S2, 100 cycle sequencing kit (Illumina). Each library was sequenced to an average raw depth of 25-30 M reads. Base calling was done by Illumina RTA3 and output of NCS was demultiplexed and converted to FastQ format with Illumina Bcl2fastq v1.9.0.

Generated, raw, 50 bp paired-end reads were trimmed via *cutadapt* [28] and *Trim Galore!* (http://www.bioinformatics.babraham.ac.uk/projects/trim_galore/). Quality control was performed using *FastQC* [29] and *MultiQC* [30]. Trimmed reads were aligned to hg38 genome assembly, indexed to GENCODE [31] (vM16) GFF3 annotation via *STAR* [32] aligner with flag ‘--quantMode GeneCounts’ for feature counting. Reverse-stranded, gene-level counts extracted from STAR output files were constructed into an experimental read count matrix using R. Low count genes (< 1 count/sample average) were filtered prior to *DESeq2* [33, 34] count normalization and subsequent differential expression analysis. Calculated differential expression probabilities were corrected for multiple testing by independent hypothesis weighting [35] for downstream analysis. A significance threshold of FDR < 0.001 was used for differentially expressed genes. Hallmark pathways and Gene Ontology Biological Processes (GOBP) gene sets were retrieved from MSigDB [36]. *ClusterProfiler* [37] was used to compute and visualize pathway enrichment of GOBP gene sets to respective gene universes, detailing the significance of the enrichment and number of genes involved previously described [38–41]. *eulerr* [42] was used to produce proportional Euler diagrams. R [43] and GraphPad Prism 9 software were used for many applications. EC cell line mutation data was obtained from the Cancer Cell Line Encyclopedia [44].

## Results and discussion

### PIK3CA mutation dramatically alters gene expression response to insulin

Of the 28 primary EC cell lines characterized by the Cancer Cell Line Encyclopedia [44], all cell lines harbor mutations in either ARID1A or TP53 (Supplemental Fig. 1). ARID1A and TP53 mutations are not observed in the normal endometrium [4, 10] and, in mice, heterozygous loss of either of these genes is sufficient for endometrial tumorigenesis in the context of co-occurring PIK3CA mutation [39, 45]. Therefore, given presence of pre-existing mutations, EC cell lines cannot be used to accurately model the phenotypic effects of acute mutation induction in cancer driver genes. Alternatively, the 12Z cell line are immortalized cells derived from ectopic endometriosis [25] and represent authentic endometriosis cells [46]. 12Z cells are not known to harbor cancer driver mutations, and they have been used to study the phenotypic effects of genetic mutations [38–40, 47–49]. Therefore, to determine the effects of genetic mutation and insulin signaling on endometrial cells, we utilized the 12Z cell model [25].

**Fig. 1:**
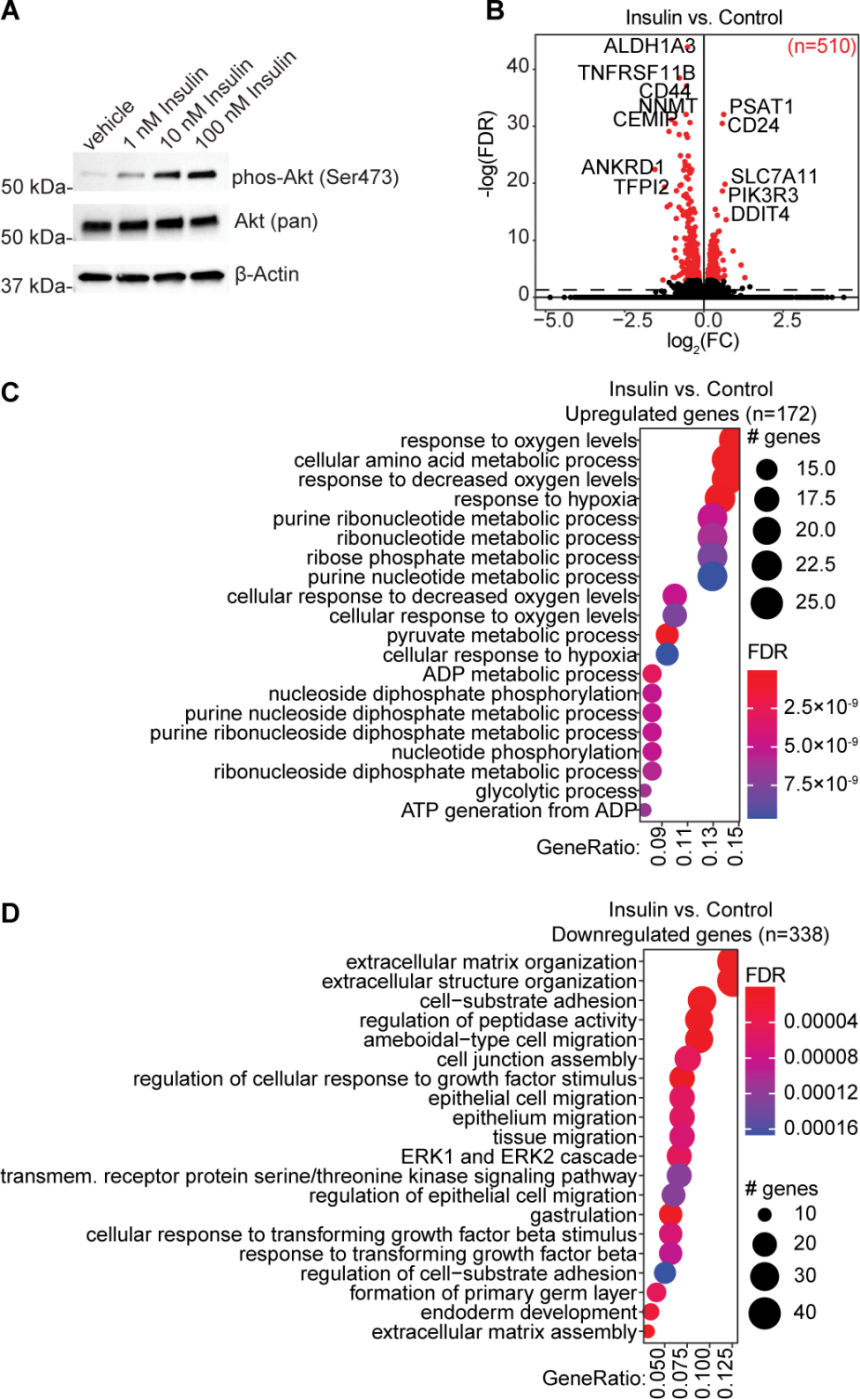
Insulin treatment of endometriotic epithelial cells results in differential gene expression. **A**, Western blot of dose response of 12Z cells to insulin for phospho-AKT (Serine 473), AKT and β-Actin. Uncropped images are available in Supplemental Fig. 2. **B**, Volcano plot of DGE in 12Z cellstreated with 100 nM insulin vs. vehicle. *FDR* < 0.001 significant genes are red. **C-D**, GOBP enrichment among genes upregulated (C) or downregulated (D) following insulin treatment

12Z cells were serum-starved and treated with doses of insulin between 1 nM and 100 nM, and AKT phosphorylation was observed in a dose-dependent manner (Fig. 1A), validating the model system. We performed RNA-seq on 12Z cells treated with 100 nM insulin and found 510 significant (FDR < 0.001) differentially expressed genes (DEG) following insulin treatment (Fig. 1B). There were 172 genes with increased expression following insulin treatment, and we examined which GOBP terms were enriched among these genes. Pathways related to oxygen levels and ribonucleotide metabolism were upregulated in 12Z cells following insulin treatment (Fig. 1C). Similarly, there were 338 genes downregulated following insulin treatment, and these genes were enriched for pathways related to extracellular matrix organization and cell adhesion, among others (Fig. 1D).

**Fig. 2:**
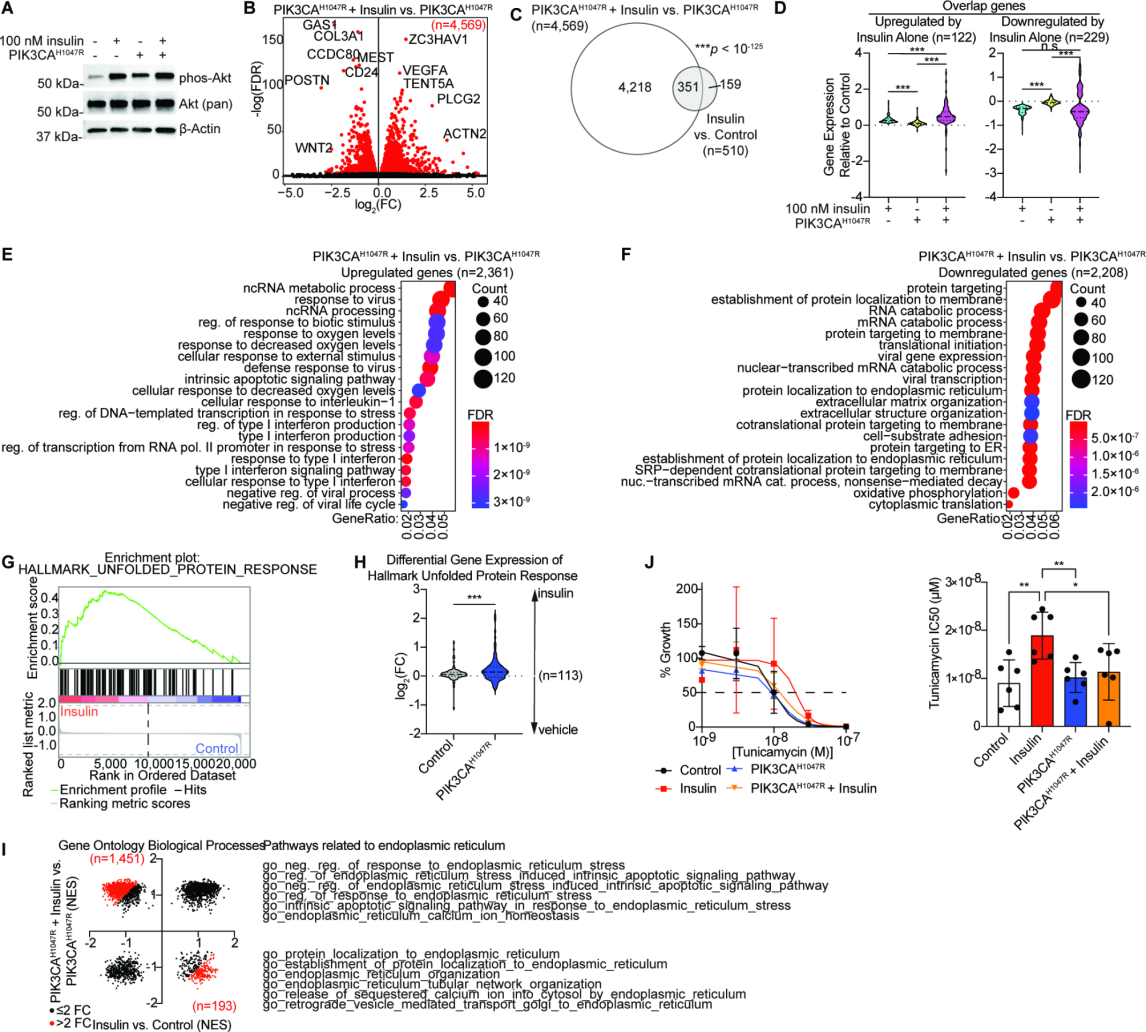
Overexpression of a mutated PIK3CA alters the response of endometrotic epithelial cells to insulin. **A**, Western blot of 12Z cell expression of phospho-AKT (Serine 473), AKT and β-Actin following PIK3CA^H1047R^ expression and insulin treatment. Uncropped images are available in Supplemental Fig. 2. **B**, Volcano plot of DGE in PIK3CA^H1047R^ 12Z cells treated with 100 nM insulin relative to vehicle, *FDR* < 0.001. Significant genes red. **C**, Euler diagram of overlapping DEG in PIK3CA^H1047R^ + Insulin vs. PIK3CA^H1047R^ and Insulin vs. Control. Statistic is hypergeometric enrichment. **D**, Violin plot of fold-change values relative to control for Insulin, PIK3CA^H1047R^ and PIK3CA^H1047R^ + Insulin for overlapping genes from F which are upregulated (n = 122, left) or downregulated (n = 229, right) in Insulin vs. Control. Statistic is paired *t*-test. **E-F**, GOBP enrichment among genes upregulated (E) or downregulated (F) in PIK3CA^H1047R^ + Insulin vs. PIK3CA^H1047R^. **G**, GSEA plot of enrichment of Insulin vs. Control datasets for Hallmark UPR. **H**, Fold-change values for Hallmark UPR genes following insulin treatment in control or PIK3CA^H1047R^ cells. Statistic is paired *t*-test. **I**, Normalized Enrichment Scores (NES) from GSEA for GOBP, Insulin vs. Control (x-axis) or PIK3CA^H1047R^ + Insulin vs. PIK3CA^H1047R^ (y-axis). Pathways with an absolute difference > 2 are red. Pathways related to ER are listed which are upregulated in PIK3CA^H1047R^ + Insulin vs. PIK3CA^H1047R^ (top) or for Insulin vs. Control (bottom). **J**, Tunicamycin dose response in Insulin, PIK3CA^H1047R^, PIK3CA^H1047R^ + Insulin or control cells (left) and IC50 values (right). Statistic is unpaired *t*-test. *p < 0.05, **p < 0.01, ***p < 0.001. Western blots are representative of n = 2 independent experiments

Given the shared roles for hyperinsulinemia and PIK3CA mutation in activating the PI3K-AKT-mTOR pathway, [12, 50], we next explored the role of PIK3CA mutation in response to insulin signaling. The most frequently mutated amino acid residue of PIK3CA in EC is histidine 1047, with the most common substitution being arginine (H1047R)[51]. This mutation is an activating mutation in the kinase domain [52] and is commonly observed in the normal endometrium [4, 10]. 12Z cells were transfected with PIK3CA^H1047R^ OE plasmid, with or without insulin co-treatment. As expected, PIK3CA^H1047R^ expression increased AKT phosphorylation, but an additive effect of insulin treatment and PIK3CA^H1047R^ expression on phos-AKT was not observed (Fig. 2A). We performed RNA-seq on 12Z cells following these treatments and considered the effects of PIK3CA^H1047R^ OE on the response to insulin treatment. PIK3CA^H1047R^ cells displayed significant (FDR < 0.001) differential expression of 4,569 genes upon insulin treatment (Fig. 2B), a roughly 9-fold increase in the number of genes affected by insulin signaling (Fig. 1B). Most of the genes affected by insulin in the control cells were also affected in PIK3CA^H1047R^ cells (68.8%), while an additional 4,218 genes were also differentially expressed in this context (Fig. 2C). These results suggest PIK3CA^H1047R^ expression is a major determinant of differential response to insulin in the endometrial epithelial cells. Among genes affected by insulin in either control cells or PIK3CA^H1047R^ cells, those upregulated by insulin in control cells were upregulated to a greater degree in PIK3CA^H1047R^ cells treated with insulin, in contrast to downregulated genes in this group (Fig. 2D). Genes upregulated by insulin in PIK3CA^H1047R^ cells were enriched for ncRNA metabolic process, response to virus and interferon production (Fig. 2E), while genes downregulated by insulin in PIK3CA^H1047R^ cells were enriched for protein targeting and localization to ER (Fig. 2F).

Both PIK3CA and insulin signaling have been shown to regulate ERS through the UPR pathway [53], so we examined the Hallmark UPR pathway and observed upregulation of this pathway in cells responding to insulin (Fig. 2G). However, the fold-change in UPR pathway genes in response to insulin was greater in PIK3CA^H1047R^-expressing cells (Fig. 2H). A more specific analysis of pathways related to ER processes revealed that insulin treatment in control cells upregulates ER organization and ER protein localization in control cells, while insulin treatment in PIK3CA^H1047R^-OE cells downregulates these pathways (Fig. 2I). Similarly, pathways related to negative regulation of ER stress and ER stress-induced apoptosis were upregulated in PIK3CA^H1047R^-expressing cells treated with insulin but downregulated in control cells treated with insulin (Fig. 2I). Next, we tested whether differences in cell growth under conditions of ER stress could be observed between these conditions through the additional activation of ER stress through tunicamycin treatment. We observed a resistance to tunicamycin in control cells treated with insulin, but insulin treatment did not afford this resistance to PIK3CA^H1047R^-expressing cells (Fig. 2J). These results suggest that, while cells normally activate the UPR upon insulin signaling, the magnitude of gene expression change, specific pathways and sensitivity to ER stress are altered in PIK3CA^H1047R^ cells.

### Overexpression of Viperin/RSAD2 is necessary for response of PIK3CA mutant cells to insulin

**Fig. 3:**
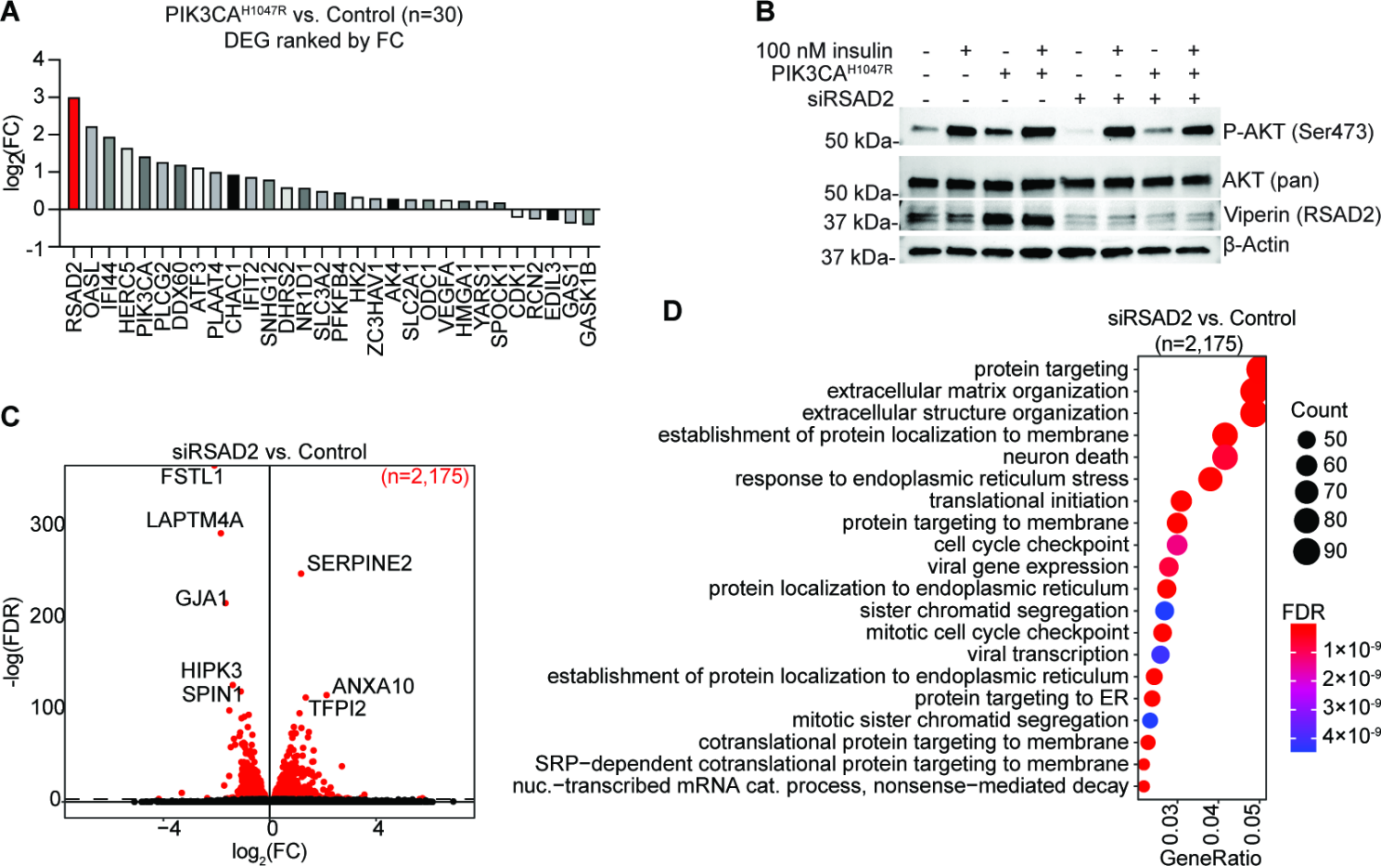
PIK3CA ^H1047R^ results in upregulation of RSAD2. **A**, Ranking of DEG in PIK3CA^H1047R^ vs. control dataset (n = 30) based on fold-change value. Gene ranked first is labeled in red. **B**, Western blot of 12Z cell expression of Viperin, phospho-AKT, AKT and β-Actin following siRSAD2, PIK3CA^H1047R^ and insulin treatment. Western blots are representative of n = 2 independent experiments. Uncropped images are available in Supplemental Fig. 2. **C**, Volcano plot of DGE in siRSAD2 vs. control, *FDR* < 0.001. Significant genes are labeled in red. **D**, GOBP enrichment among genes differentially expressed in siRSAD2 vs. control dataset

**Fig. 4:**
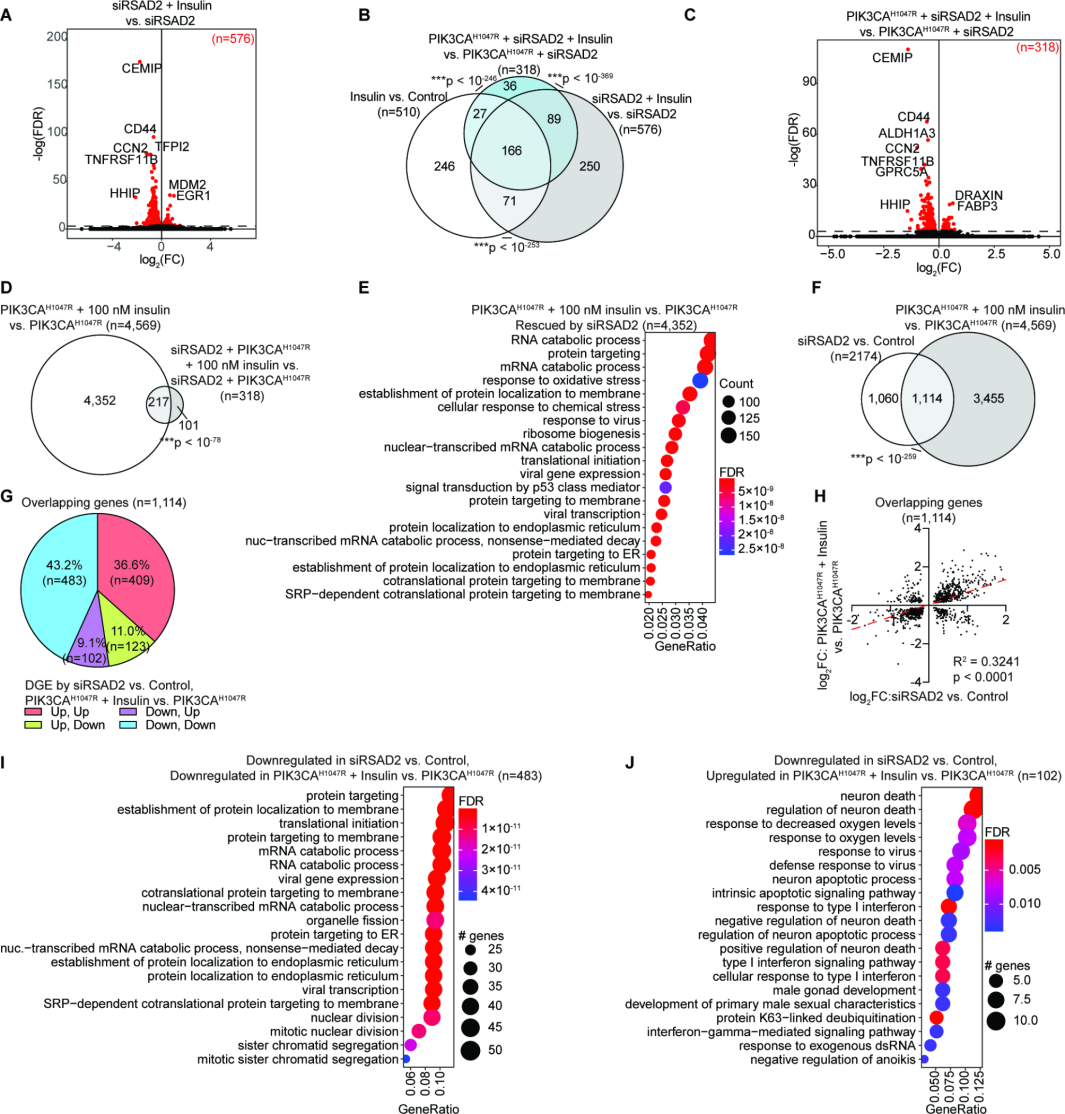
Knockdown of RSAD2 rescues DGE observed in insulin + PIK3CA ^H1047R^. **A**, Volcano plot of DGE following insulin treatment in siRSAD2 cells relative to vehicle, *FDR* < 0.001. Significant genes are labeled in red. **B**, Euler diagram, overlapping DEG in Insulin vs. Control, siRSAD2 + Insulin vs. siRSAD2 and siRSAD2 + PIK3CA^H1047R^ + Insulin vs. siRSAD2 + PIK3CA^H1047R^. Statistic is hypergeometric enrichment. **C**, Volcano plot of DGE following insulin treatment in siRSAD2 and PIK3CA^H1047R^ cells relative to vehicle, *FDR* < 0.001. Significant genes red. **D**, Euler diagram of overlapping DEG in PIK3CA^H1047R^ + Insulin vs. PIK3CA^H1047R^ and siRSAD2 + PIK3CA^H1047R^ + Insulin vs. siRSAD2 + PIK3CA^H1047R^. Statistic is hypergeometric enrichment. **E**, GOBP enrichment among genes differentially expressed in PIK3CA^H1047R^ + Insulin vs. PIK3CA^H1047R^ but not siRSAD2 + PIK3CA^H1047R^ + Insulin vs. siRSAD2 + PIK3CA^H1047R^. **F**, Euler diagram of overlapping DEG in PIK3CA^H1047R^ + Insulin vs. PIK3CA^H1047R^ and siRSAD2 vs. Control. Statistic is hypergeometric enrichment. **G**, Pie chart displaying direction of DGE for overlapping genes from J, and directionality in siRSAD2 vs. Control (listed first) and PIK3CA^H1047R^ + Insulin vs. PIK3CA^H1047R^ (listed second). **H**, Fold-change values for overlapping genes from J in siRSAD2 vs. Control (x-axis) and PIK3CA^H1047R^ + Insulin vs. PIK3CA^H1047R^ (y-axis). Red dashed line is line of best fit. Statistic is Pearson’s correlation. **I**, GOBP enrichment among overlapping genes downregulated in both datasets (n = 483) as in K. **J**, GOBP enrichment among overlapping genes downregulated in siRSAD2 vs. Control and upregulated in PIK3CA^H1047R^ + Insulin vs. PIK3CA^H1047R^ (n = 102) as in K

We next wanted to characterize the mechanism by which PIK3CA^H1047R^ alters response to insulin. We examine the genes which were differentially expressed upon PIK3CA^H1047R^ OE alone relative to control cells and observed differential gene expression (DGE) of only 30 genes. The gene with the greatest absolute fold-change following PIK3CA^H1047R^ expression was RSAD2 (Fig. 3A). RSAD2 is a radical S-adenosylmethionine (SAM) domain-containing protein also called Viperin, which can inhibit viral replication and is usually induced by interferons or directly by viruses [23]. Upregulation of Viperin by PIK3CA^H1047R^ was confirmed by western blot (Fig. 3B). To explore the role of RSAD2 in endometriotic epithelial cells in several context, we transfected 12Z cells with siRNA targeting RSAD2 (siRSAD2) and confirmed knockdown by western blot (Fig. 3B). By RNA-seq, knockdown of RSAD2 resulted in significant DGE of 2,175 genes (Fig. 3C). These 2,175 genes were enriched for protein targeting, protein localization and extracellular matrix pathways (Fig. 3D). Following knockdown of RSAD2, insulin treatment resulted in a DGE of 576 genes (Fig. 4A), comparable to the number affected by insulin treatment in control cells (Fig. 1B). The DGE induced by insulin in siRSAD2 cells significantly overlapped with DGE in control cells treated with insulin (Fig. 4B). When we knocked down expression of RSAD2 in PIK3CA^H1047R^ cells and then treated with insulin, we observed DGE of 318 genes (Fig. 4C), which is only 7.2% the number of genes affected in PIK3CA^H1047R^ cells treated with insulin, suggesting a rescue of the effect observed following the combination of PIK3CA^H1047R^ OE and insulin. Most of these 318 genes were also affected by insulin in PIK3CA^H1047R^ cells (Fig. 4D). The 4,352 genes rescued by RSAD2 knockdown were enriched for RNA catabolism, protein targeting and response to virus pathways (Fig. 4E). Since RSAD2 knockdown alone affected gene expression, we asked whether RSAD2 knockdown affects any of the same genes affected by insulin treatment of PIK3CA^H1047R^ cells and observed a significant overlap (Fig. 4F). Surprisingly, the direction of gene expression change was the same for most these genes (79.8%, Fig. 4G,H), meaning that knockdown of RSAD2 in control cells in some cases had the same effect as insulin treatment in PIK3CA^H1047R^ cells for 892 of these 1,114 genes. Among these, genes which were downregulated in both cases were enriched for protein targeting and translation (Fig. 4I), while genes downregulated by siRSAD2 in control cells but upregulated by insulin in PIK3CA^H1047R^ cells were enriched for neuron death and response to virus pathways (Fig. 4J).

### Viperin is necessary but not sufficient for altered insulin response

**Fig. 5:**
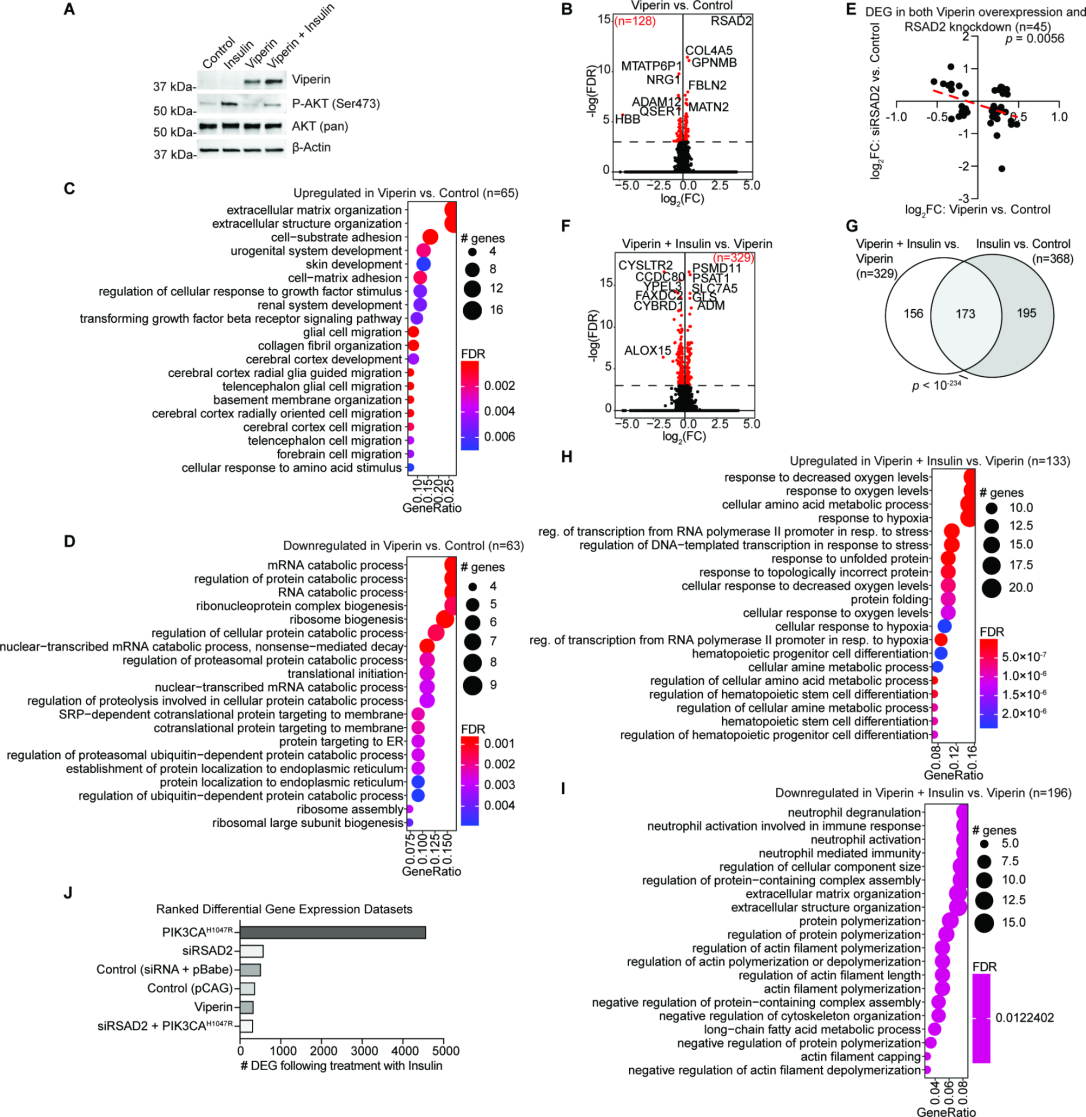
Overexpression of Viperin is insufficient for DGE observed in insulin + PIK3CA ^H1047R^. **A**, Western blot of 12Z cell expression of Viperin, phospho-AKT, AKT and β-Actin following Viperin OE and insulin treatment. Western blots are representative of n = 2 independent experiments. Uncropped images are available in Supplemental Fig. 2. **B**, Volcano plot of DGE in 12Z cells with Viperin OE vs. control, *FDR* < 0.001. Significant genes are labeled in red. RSAD2 -log(FDR) value is out of scale with the y-axis for visualization purposes. **C**, GOBP enrichment among genes upregulated following Viperin OE. **D**, GOBP enrichment among genes downregulated following Viperin OE. **E**, Correlation between fold-change values for overlapping genes among Viperin vs. Control and siRSAD2 vs. Control datasets (n = 45). RSAD2 expression was removed from the analysis. Red dashed line is line of best fit. Statistic is Pearson’s correlation. **F**, Volcano plot of DGE in 12Z cells treated with Viperin OE and Insulin vs. Viperin OE alone, *FDR* < 0.001. Significant genes are labeled in red. **G**, Euler diagram of overlapping DEG in Viperin + Insulin vs. Viperin and Insulin vs. Control (pCAG). Statistic is hypergeometric enrichment. **H-I**, GOBP enrichment among genes upregulated (H) or downregulated (I) in insulin-treated Viperin overexpressing cells. **J**, Number of DEG by insulin in each cell context. Datasets ranked by number of DEG

We next tested whether Viperin OE was sufficient to induce DGE upon insulin treatment. We utilized a previously published Viperin OE vector [27] to overexpress Viperin in 12Z cells (Fig. 5A). Viperin OE resulted in DGE of 128 genes, with RSAD2 being the most significant (Fig. 5B). Upregulated genes were enriched for extracellular matrix organization and cell adhesion pathways (Fig. 5C) while downregulated genes were enriched for catabolic processes and ribosome biogenesis (Fig. 5D). Among other genes which were affected by both siRSAD2 and Viperin OE, we found a negative correlation in the fold-change values of these genes, suggesting consistency between the models (Fig. 5E). Upon stimulation with insulin, cells with Viperin OE differentially expressed 329 genes (Fig. 5F), although most were the same genes affected by insulin stimulation in control cells (Fig. 5G). The reduced number of total genes differentially expressed upon insulin treatment in the Viperin OE cells may be related to a reduction in AKT phosphorylation in this condition (Fig. 5A). Genes upregulated by insulin in the context of Viperin OE were enriched for pathways related to response to oxygen, metabolic process and ERS (Fig. 5H), while downregulated genes were enriched for pathways related to neutrophil response (Fig. 5I). These results suggest that Viperin is necessary, but not sufficient, for the increased DGE observed in during insulin treatment of PIK3CA^H1047R^ cells, as knockdown of RSAD2 can rescue this effect but Viperin OE is not sufficient to recreate this phenotype (Fig. 5J).

### Insulin-induced, viperin dependent, PIK3CA ^H1047R^ specific gene expression profile is related to interferon signaling and viral response

**Fig. 6:**
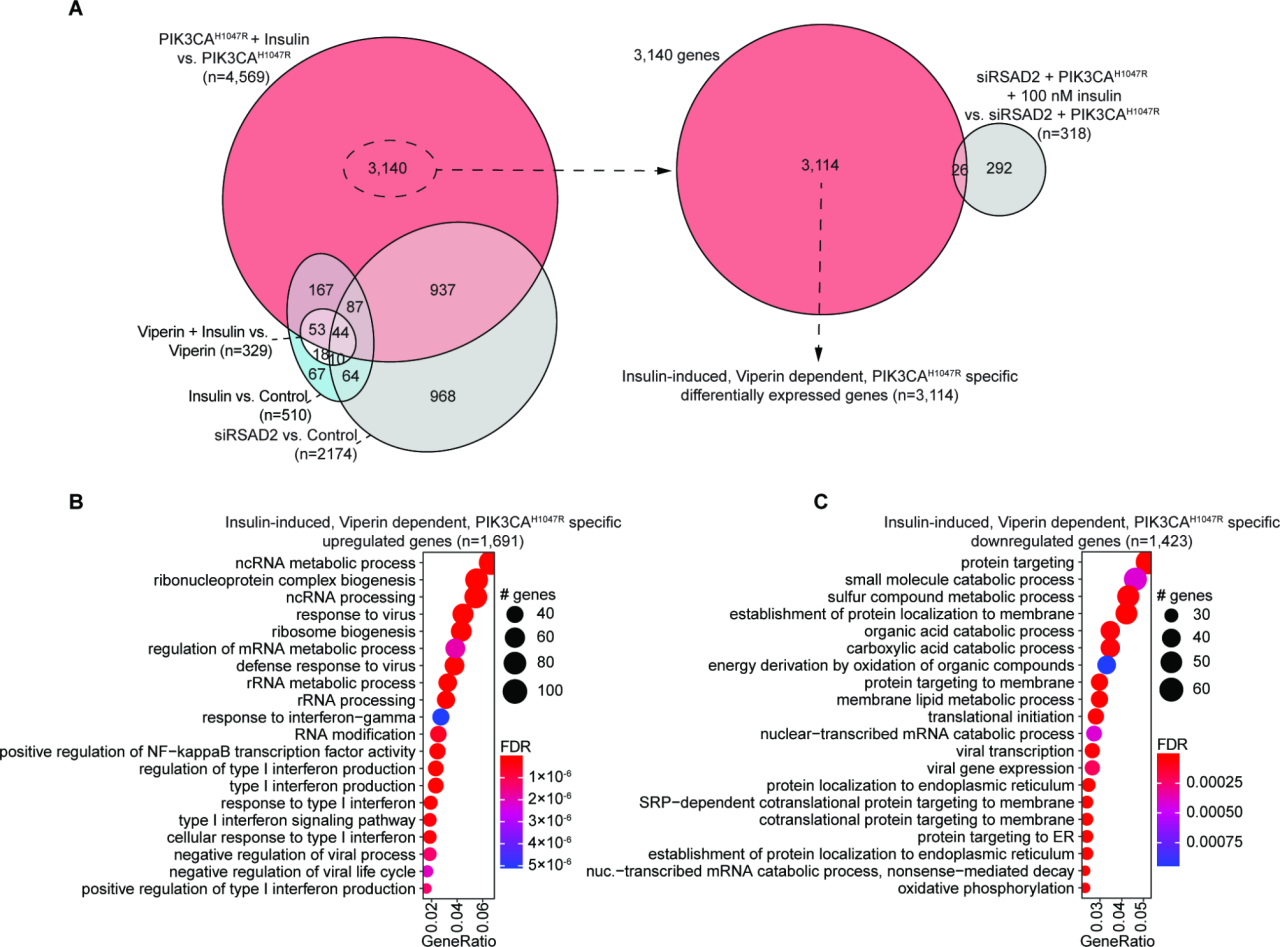
Identification of an Insulin-induced, Viperin-dependent, PIK3CA ^H1047R^-specific DEG. **A**, Euler diagrams demonstrating the Insulin-induced, Viperin-dependent, PIK3CA^H1047R^-specific geneset. **B-C**, GOBP enrichment among genes upregulated (B) or downregulated (C) among geneset from A

Utilizing the data generated from control cells treated with insulin, Viperin OE cells treated with insulin, cells with RSAD2 knockdown and cells with both PIK3CA^H1047R^ and siRSAD2 treated with insulin, we isolated a list of 3,114 genes which are induced by insulin treatment, dependent on Viperin expression and specific to PIK3CA^H1047R^ cells (Fig. 6A). Of these 3,114 genes, upregulated genes were enriched for non-coding RNA processing, ribonucleoprotein complex biogenesis, response to virus and interferon production (Fig. 6B), while downregulated genes were enriched for protein targeting to the ER, catabolic processes and viral transcription (Fig. 6C). These results suggest that in PIK3CA^H1047R^ cells, insulin treatment results in DGE of genes related to interferon signaling, viral response, catabolism and protein targeting, which depend on Viperin.

## Conclusion

In the present study we identified a transcriptomic response to insulin that is altered when PIK3CA mutations are present in the endometriotic epithelium. We determined that these gene expression changes were due in part to expression of Viperin, which is necessary but not sufficient for this response. Although Viperin has been understood as an interferon-induced antiviral enzyme with radical S-adenosylmethionine activity for decades, it was only recently uncovered that the mechanism of action for Viperin lies in the conversion of CTP to an alternate nucleotide, ddhCTP (3′-deoxy-3′,4′-didehydro-CTP) [54]. ddhCTP inhibits viral replication by acting as a chain terminator during viral RNA replication without impacting host DNA and RNA replication. Viperin has been shown to be overexpressed in the endometrium during early pregnancy in several animal models [24, 55, 56]. A screen of genes involved in EC prognosis identified high RSAD2 gene expression as being associated with decreased survival probability [57]. Our results suggest that mutant PIK3CA in alters the response of endometriotic epithelial cells to the direct action of insulin, and that this differential response is promoted by the overexpression of Viperin. Future studies will focus on the role of Viperin in EC tumor microenvironment, to determine the impact of Viperin OE during hyperinsulinemia and subsequent interferon production on tumor progression.

## Electronic supplementary material

Below is the link to the electronic supplementary material.

Supplementary Material 1

## Data Availability

RNA-seq data is available at GEO accession series GSE215260 with reviewer token ixgtkqmkvzmdhcx.
