# Supplementary material for: PIK3CA mutation in endometriotic epithelial cells promotes viperin-dependent inflammatory response to insulin
Source: Reprod Biol Endocrinol. 2023 May 11;21:43. doi: 10.1186/s12958-023-01094-6 (PMC10173629; doi:10.1186/s12958-023-01094-6)
Supplement: Supplementary file 1 — Supplementary Material 1 [file 12958_2023_1094_MOESM1_ESM.pdf]

PIK3CA mutation in endometriotic epithelial cells promotes viperin-dependent inflammatory response to insulin

Wilson et al. 2023

Supplemental Figure

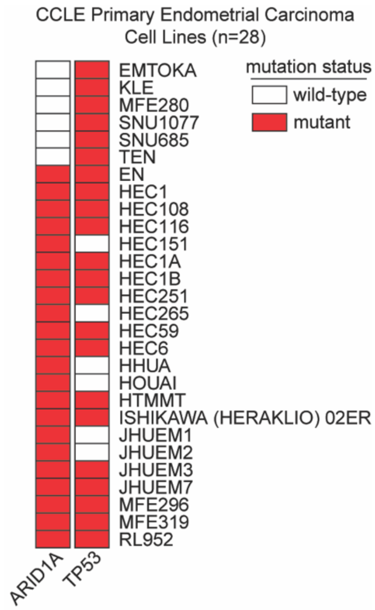

Supplemental Figure 1. ARID1A and TP53 mutation profiles of endometrial cancer cell lines. Mutation data was collected from the Cancer Cell Line Encyclopedia (CCLE) for 28 primary endometrial cancer cell lines. Red indicates a mutation was observed for ARID1A (left column) or TP53 (right column). Cell lines were clustered based on ARID1A mutation status.

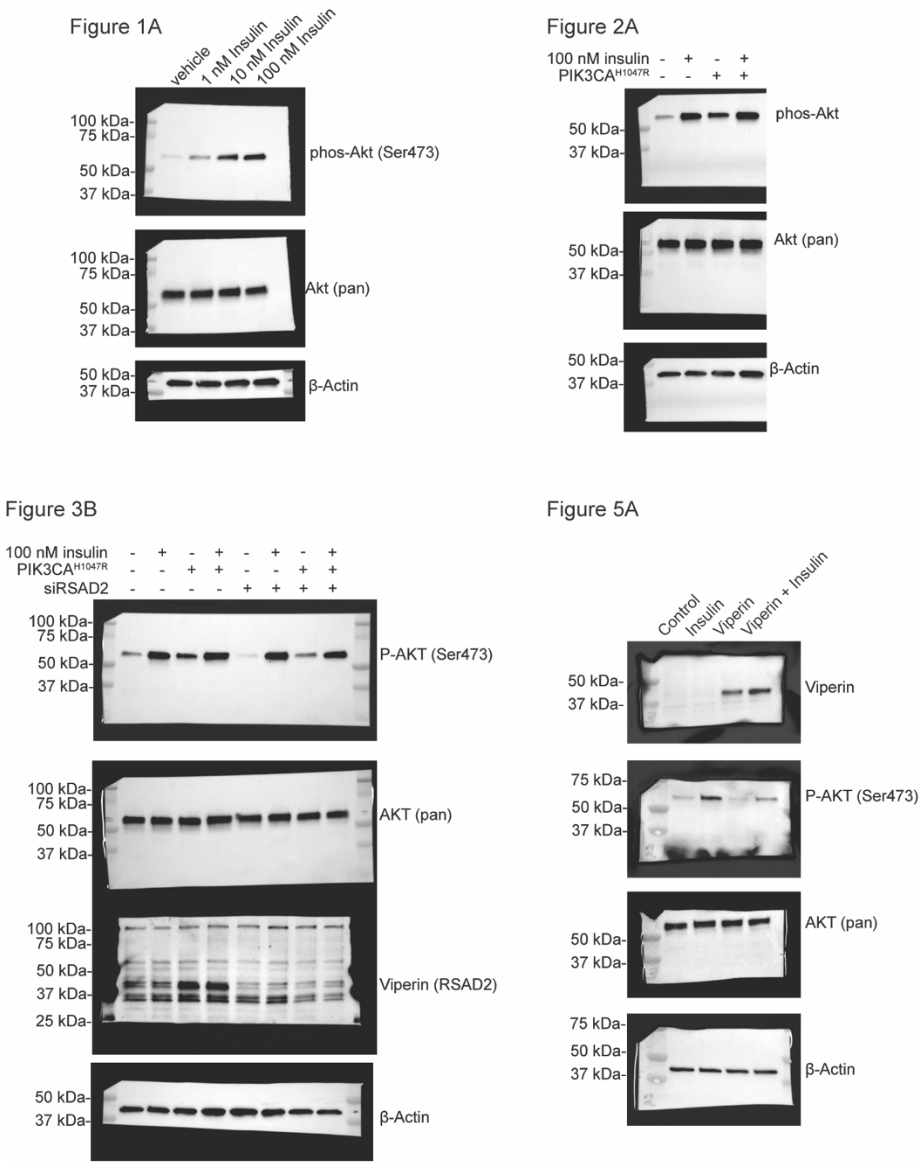

Supplemental Figure 2. Uncropped western blots.
